# The varied functions of aluminium-activated malate transporters–much more than aluminium resistance

**DOI:** 10.1042/BST20160027

**Published:** 2016-06-09

**Authors:** Antony J. Palmer, Alison Baker, Stephen P. Muench

**Affiliations:** * School of Biomedical Sciences, Faculty of Biological Sciences, University of Leeds, Leeds LS2 9JT, U.K.; † Astbury Centre for Structural Molecular Biology, University of Leeds, Leeds LS2 9JT, U.K.; ‡ Centre for Plant Science, University of Leeds, Leeds LS2 9JT, U.K.; § School of Molecular and Cellular Biology, Faculty of Biological Sciences, University of Leeds, Leeds LS2 9JT, U.K.

**Keywords:** aluminium-activated malate transporter (ALMT), aluminium resistance, γ-aminobutyric acid (GABA), ion channel, malate, stomata

## Abstract

The ALMT (aluminium-activated malate transporter) family comprises a functionally diverse but structurally similar group of ion channels. They are found ubiquitously in plant species, expressed throughout different tissues, and located in either the plasma membrane or tonoplast. The first family member identified was *Ta*ALMT1, discovered in wheat root tips, which was found to be involved in aluminium resistance by means of malate exudation into the soil. However, since this discovery other family members have been shown to have many other functions such as roles in stomatal opening, general anionic homoeostasis, and in economically valuable traits such as fruit flavour. Recent evidence has also shown that ALMT proteins can act as key molecular actors in GABA (γ-aminobutyric acid) signalling, the first evidence that GABA can act as a signal transducer in plants.

## Introduction

The aluminium-activated malate transporter (ALMT) family is found ubiquitously in sequenced genomes throughout the plant kingdom [1] and was named when the first member of the family to be discovered was found to be involved in aluminium resistance in wheat [2]–although proteins of this family are channels, rather than transporters as their name suggests. The first characterized homologue in *Arabidopsis thaliana*, *At*ALMT1 [3], was similarly found to be involved in Al-resistance. However, in addition to ALMT1, the gene family in this species contains 13 other members suggesting that they are involved in more than just Al-resistance, and some of these have already shown to have a wide range of other roles [4]. To date, the ALMT family has been shown to be central to physiological processes such as control of stomatal aperture [5,6] and anion homoeostasis [7]. Furthermore, increasing attention is being paid due to their potential role in economically valuable traits such as fruit flavour [8] and grain filling [9], and more recently they have been shown to be key mediators of GABA (γ-aminobutyric acid) signalling [10]. Identification of the molecular actors in these processes is already helping guide marker-assisted breeding [11]. The ALMT family is now known to be central to many physiological processes, with scope for even more diversity as many homologues are still to be characterized and thus, a comprehensive review of all currently known functions is a timely addition to the literature.

## Aluminium resistance

### Overcoming aluminium toxicity on acid soils

Acid soils are prevalent worldwide, comprising around half of all potentially arable land [12]. In these soils, aluminium ions become solubilized and damage crops via root growth inhibition [13] and, to compound the problem, nutrients such as phosphate become less available [14]. Several plant species have been identified as aluminium-resistant and they use a variety of mechanisms including thickening of cell walls [15], active transport of aluminium away from sensitive organs [16], or, prominently, organic acid exudation, chiefly either by release of malate or citrate [17,18].

Studies on crosses of near isogenic Al-resistant and Al-sensitive wheat (*Triticum aestivum*) cultivars alongside electrophysiological studies using *Xenopus* oocytes [19,2,20] first identified *Ta*ALMT1 as the channel responsible for malate exudation from root tips, providing the primary mechanism of aluminium resistance. *Ta*ALMT1 is constitutively expressed in the root apices of Al-resistant wheat and malate chelates Al^3+^ forming a 2:2 complex with the trivalent aluminium ions, thus encasing the ions and rendering them non-toxic [21]. This allows longer root growth and greater yields compared with a sensitive cultivar grown on acid soils. In addition to protection from Al^3+^ toxicity, malate extrusion has the benefit of increasing phosphate availability in the soil–since Al^3+^ binds and complexes phosphate [22]. This is part of a host of processes activated in plants for improved phosphorous usage [14,23]. Heterologous expression of *Ta*ALMT1 in cultured tobacco cells, *Xenopus* oocytes, and transgenic rice plants has shown efflux of malate activated by the presence of Al^3+^ and expression confers Al-resistance to tobacco cells [2]. Importantly, transgenic expression of *Ta*ALMT1 in Al-sensitive barley plants rendered them resistant to aluminium toxicity [24]. In one study, transgenic plants grown on acid soil displayed root growth similar to that seen in neutral soils and a doubling in yield when expressing just the single gene [23], making it a powerful tool for transgenic crop development. A thorough review of transgenic approaches using ALMT1 and other genes can be found in Ryan et al. [25].

### Other ALMTs involved in aluminium resistance

**Figure 1:**
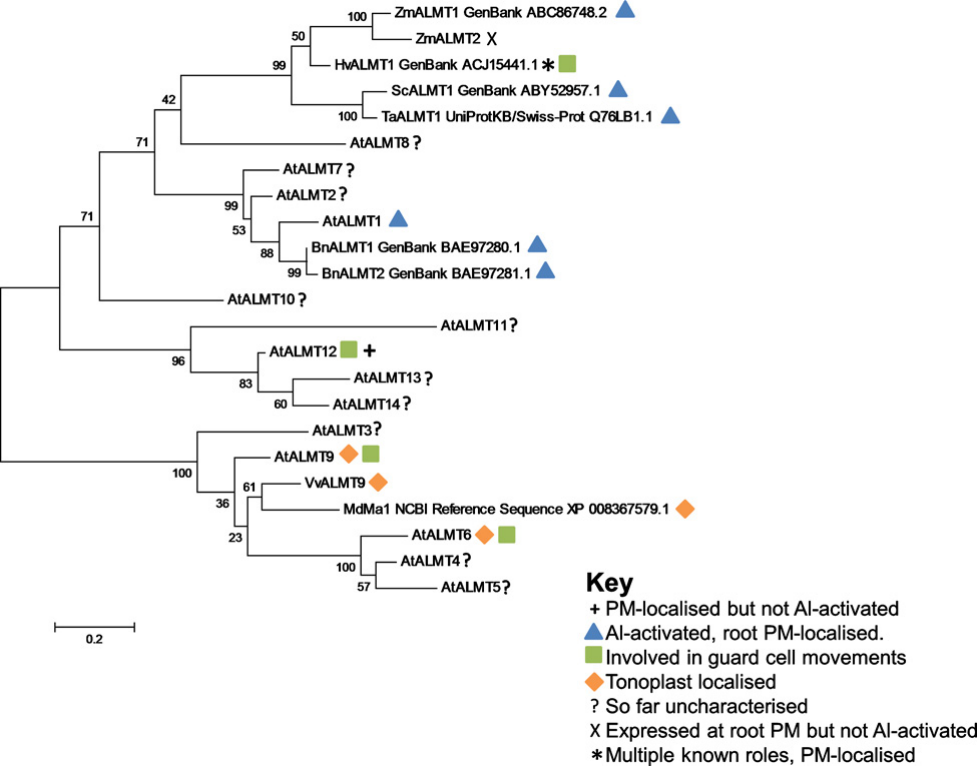
Phylogenetic tree of ALMTs with known functions plus family members from *Arabidopsis* with unknown function Numbers on nodes denote likelihood of correctness. Tree made using MEGA6 software [51], sequences were aligned by the inbuilt MUSCLE functionality, and constructed with the maximum likelihood method and 500 iterations of bootstrapping.

Since the characterization of *Ta*ALMT1, several ALMTs from other species including oilseed rape, *Arabidopsis*, rye, soybean and Yorkshire fog have been shown to be vital for Al-resistance and characterized (see Figure 1 and Table 1). These channels are activated by Al^3+^ and in some cases their expression is also up-regulated upon sensing Al^3+^ [26].

*At*ALMT1 is a malate channel critical for aluminium resistance in *Arabidopsis*, expressed at the plasma membrane of the epidermal cells of the root tip [3]. This protein shows 41% sequence identity and 63% similarity to *Ta*ALMT1. However, in contrast, *At*ALMT1 is not constitutively expressed; instead, expression is up-regulated by aluminium [26,27], mediated by the transcription factors STOP1, STOP2 and WRKY, which also regulate other genes critical for tolerance of acid soils [28–30]. In addition, two genes with 95% sequence identity with one another were identified in oilseed rape (*Brassica napus)* [31,32], as well as *Gm*ALMT1 in soybean (*Glycene max*), and *Sc*ALMT1 in rye (*Secale cereale*) [33,34]. These genes are expressed at root tip plasma membranes, and the corresponding proteins are permeable to malate and are activated by aluminium, showing this mechanism for Al-resistance is widespread among plant species. In addition, *Gm*ALMT1 channel activity has also been shown to be regulated by pH changes and phosphorous concentration [35]. Yorkshire fog (*Holcus lanatus*) *Hl*ALMT1 has also been identified as an important resistance gene in the model grass species, and has Al-activated expression similar to *At*ALMT1 controlled by a Al-responsive transcription factor ART1, with expression levels in different accessions controlled by the number of binding regions for ART1 in the promoter region [36].

### Other root-related functions

*Zm*ALMT2 from maize (*Zea mays*) has been shown to be root localized and to release malate into the soil. Unlike *At*ALMT1 and *Ta*ALMT1, however, this is not correlated with Al-resistance, but instead is likely to provide solubilization for soil nutrients, such as phosphate, as discussed above for *Ta*ALMT1. As it is also found in vascular tissue, *Zm*ALMT2 could also play a role in the transport of organic acids or mineral anions in the xylem [37].

## Guard cell movements

Another vital role played by some ALMT family members is as molecular components of the guard cell movements that regulate gas exchange across leaf surfaces. Plants control CO_2_ uptake and water loss by regulating the aperture of the stomatal pores. Three family members from *Arabidopsis*, *At*ALMT6, *At*ALMT9 and *At*ALMT12, are involved in opening and closing of stomata, with movement driven by osmotically active inorganic and organic ions [38]. Upon stomatal opening, K^+^ enters the guard cell via voltage-gated inward rectifying potassium channels, driven by the electrochemical gradient maintained by ATP-driven proton pumping. Malate synthesis from stored starch provides a charge-balancing ion, and is taken up into the vacuole via *At*ALMT6, and similarly *At*ALMT9 acts to permit entry of chloride counterions into the vacuole. The increase in solutes draws water into the cell down the water potential gradient, swelling the cell. The process is inverted during stomatal closing. The membrane is depolarized, prompted by the action of *At*ALMT12/QUAC1 (quick anion channel 1) releasing malate rapidly, this allows K^+^ to flow out of the cell, accompanied by Cl^−^ and NO_3_^−^ anions via SLAC1 (slow anion channel 1). In addition, *At*ALMT6 activity is regulated in part by cytosolic malate concentration, and so malate can flow out of the vacuole to either be lost via *At*ALMT12/QUAC1 or used in metabolism. This loss of osmotica drives a loss of water and closing of the stomata.

**Figure 2:**
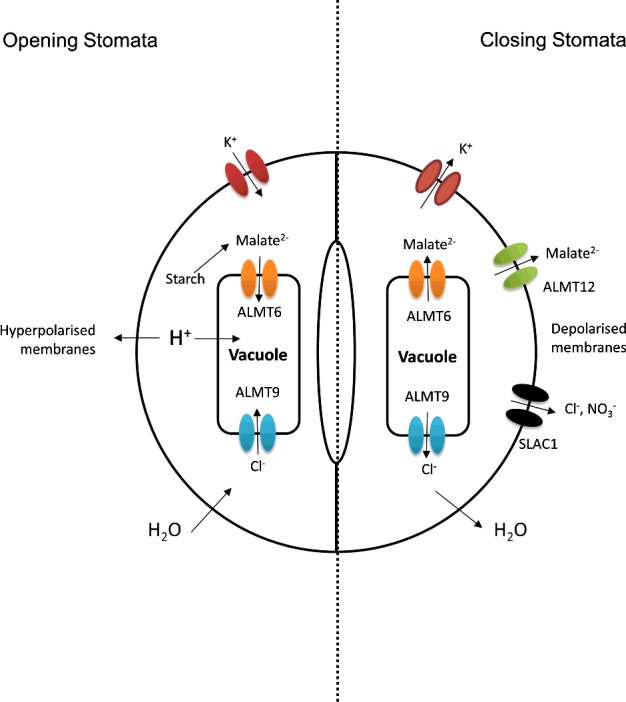
Schematic of ion currents in stomatal opening and closing K^+^ ions enter the cell during opening, and the charge is balanced by malate synthesis from starch, which is then taken up into the vacuole via ALMT6 and activates ALMT9 to permit Cl^−^ uptake into the vacuole. This increase in osmotic potential draws water into the cell, leading to swelling and stomatal opening. Stomatal closing, conversely, is driven by release of K^+^ ions, which is permitted during membrane depolarization and driven by ALMT12. Additionally, anions are released by SLAC1 over a longer period of time. Loss of osmolytes leads to concomitant loss of water and thus stomatal closing.

*At*ALMT6 is expressed in guard cell vacuoles and is a malate channel, specific for divalent malate involved in stomatal movements. It is not aluminium activated but instead is controlled by light, ABA [39], pH and cytosolic malate concentration [4]. As transport is dependent on the concentration of malate in the cytosol and the tonoplast membrane potential, *At*ALMT6 can mediate both malate uptake into and release from the vacuole in guard cells, with uptake during stomatal opening, and release during stomatal closing as shown in Figure 2. Interestingly, in *A. thaliana* expression is also seen in floral organs, suggesting another role yet to be elucidated. Knockout plants did not show phenotypic differences, indicating functional redundancy in vacuolar malate channels, perhaps from the action of *At*ALMT9 and perhaps also *At*ALMT5, which has been shown to be expressed in guard cells, and to be closely related to *At*ALMT6 (see Figure 1).

*At*ALMT9 is a vacuolar chloride channel in guard cells and *Atalmt9* knockouts show impaired stomatal opening [6]. It permits chloride to enter the vacuole, providing a charge-balance for K^+^ in the same way as malate. Malate in the cytosol (synthesized from starch) activates the channel, meaning malate can act both as an osmolyte and as a signalling molecule in guard cells. Interestingly, kinetic data suggest *At*ALMT9 is multimeric, with a number of subunits >2.5 with more recent work suggesting that it forms a tetramer [40]. Furthermore, cytosolic nucleotides such as ATP block the activity of *At*ALMT9, competing with malate for a binding site [41]. The H^+^-V-ATPases that maintain a hyperpolarized tonoplast consume ATP, provoking lowered cytosolic ATP concentrations, removing this block and thus permitting currents via *At*ALMT9 and facilitating anion uptake into the vacuole.

*At*ALMT12 (also known as QUAC1) is expressed in guard cell plasma membranes and operates as an R-type (rapid-type) channel crucial for stomatal closing [5], allowing rapid malate release. One study also found localization to endomembranes, although this is possibly an artefact of overexpression [42]. The channel opens with fast kinetics upon membrane depolarization, releasing malate into the apoplasm in parallel with K^+^ release through potassium channels to maintain the depolarization. Moreover, external malate causes increased activation of the channel and thus may represent a positive-feedback loop. Loss-of function mutants confer a wilty phenotype due to their impaired stomatal closing. Rather than being ligand gated, as seen in ALMT1, ALMT12 activity is voltage gated; however, the voltage sensor is yet to be identified, although it is likely in the CTD (C-terminal domain), which has been shown to be vital for regulation [43].

## Other roles

### Malate storage and homoeostasis

*At*ALMT9, in addition to its role in guard cells (discussed above and summarized in Table 1), has been shown to be permeable to both chloride and malate and expressed strongly throughout leaf mesophyll tissue. It is likely to have a role in homoeostasis: ensuring that the concentration of malate–which plays an essential role in metabolism as part of the citric acid cycle–remains stable within the cytoplasm. Similar to *At*ALMT6, the channel is likely to work in both directions, by storing excess in the vacuole and releasing it when required to regulate osmotic potential and C-metabolism [44].

**Table 1:** Summary of known functions and localization of ALMTs from several species

Gene	Organism	Localization	Al-activated?	Function
*At*ALMT1	*Arabidopsis thaliana*	Root cell plasma membranes	Y	Aluminium resistance
*At*ALMT6	*Arabidopsis thaliana*	Tonoplast	N	Guard cell malate currents
*At*ALMT9	*Arabidopsis thaliana*	Tonoplast	N	Chloride currents in guard cells and malate homoeostasis
*At*ALMT12	*Arabidopsis thaliana*	Plasma membrane of guard cells	N	Stomatal closing
*Ta*ALMT1	Wheat, *Triticum aestivum*	Root cell plasma membranes	Y	Aluminium resistance
*Hl*ALMT1	*Holcus lanatus*	Root plasma membrane	Y	Aluminium resistance
*Vv*ALMT9	Grape, *Vitis vinifrera*	Tonoplast of berry mesocarp	N	Fruit flavour/vacuolar malate uptake
*Md*MA1	Apple, *Malus domestica*	Tonoplast	N	Fruit flavour/vacuolar malate uptake
*Bn*ALMT1	Oilseed rape, *Brassica napus*	Root cell plasma membranes	Y	Aluminium resistance
*Bn*ALMT2	Oilseed rape, *Brassica napus*	Root cell plasma membranes	Y	Aluminium resistance
*Sc*ALMT1	Rye, *Secale cereale*	Root cell plasma membranes	Y	Aluminium resistance
*Gm*ALMT1	Soybean, *Glycine max*	Root cell plasma membranes	Y	Aluminium resistance
*Hv*ALMT1	Barley, *Hordeum vulgare*	Root cell plasma membranes and guard cells	N	Maintaining turgor in growing cells, guard cell movements
*Zm*ALMT1	Maize, *Zea mays*	Plasma membranes throughout plant	N	Inorganic anion homoeostasis
*Zm*ALMT2	Maize, *Zea mays*	Root plasma membrane	N	Constitutive malate efflux, not Al related

### Cell elongation and nutrient storage

Barley (*Hordeum vulgare*) *Hv*ALMT1 is a malate channel expressed in guard cells and the root elongation zone, as well as floral tissues and seeds [45]. Although it has the greatest sequence similarity to *Ta*ALMT1 of any barley gene and localizes to the plasma membrane, it is not involved in Al-resistance but seems to have several distinct roles within the plant. *Hv*ALMT1 over-expressing lines take longer to close their stomata [46] and RNAi knockouts show a similar phenotype to *Atamlt12* knockouts [9], so *Hv*ALMT1 is likely to be a functional homologue of *At*ALMT12. In expanding cells *Hv*ALMT1 may help provide an osmotic balance and regulate turgor. Additionally, later studies have shown that this channel plays a role in seed development during acidification of the starchy endosperm, which is required for enzyme activity [9]. Rather than directly causing acidification itself (this is probably caused by a H^+^-ATPase pumping protons from the aleurone) release of malate is suggested to act as a counterion for H^+^ and other positively charged nutrients such as K^+^, helping to maintain electroneutrality and osmotic balance in a similar manner to the role of malate in guard cell movements. The significant difference in function between *Hv*ALMT1 and *Ta*ALMT1 despite strong sequence similarity again highlights that small differences in sequence can underlie large changes in function.

### Fruit flavour

Malic acid is an important component of apple taste, grape quality and wine production: an economically significant set of traits. A malic acid channel with high homology to *At*ALMT genes has been shown to be responsible for the acidity of apples, and functions to accumulate malic acid in the vacuole [8,47]. Similarly, grape berries contain an ALMT family member expressed in the tonoplast of berry mesocarp tissue responsible for malate and tartrate accumulation [48]. Understanding of the action of these genes could be valuable for fruit and wine development [47].

### Inorganic anion homoeostasis

*Zm*ALMT1 from maize was one of the first family members to be described that did not have a role in aluminium resistance. It localizes to plasma membranes throughout the plant, but is less permeable to organic anions and instead is probably involved in inorganic anion homoeostasis and mineral nutrition [7].

### GABA signalling

A recently discovered role of ALMTs is in mediating GABA signalling in plants. This is the first evidence for GABA signalling in plants in addition to its established role as a metabolite [10]. GABA–a non-protein amino acid–accumulates in plant tissues in response to biotic and abiotic stresses, and has a central role in pollen tube growth and regulation of root growth [49]. Recently, it has been shown that GABA's influence is exerted by interaction with ALMT proteins, and a putative GABA-binding motif has been identified in the CTD. GABA binding negatively regulates ion flux through the channel, i.e. decreasing carbon flux from roots in the case of *Ta*ALMT1. Additionally, as plant anion equilibrium potentials are strongly positive and plant action potentials are generated by voltage-gated ion channels, GABA inhibition of ALMTs will hyperpolarize membranes and decrease excitability.

Moreover, to advance studies of ALMTs, perhaps parallels can be drawn with the more well-studied GABA(A) receptors from mammals, which have a greater level of structural detail elucidated already. Although they are different gene families, it may be possible to apply insights from mammalian proteins to design experiments to study ALMTs as GABA(A) receptors are predicted to have a similar overall architecture, being ∼450AA long and divided into a membrane-embedded half and a soluble half [50]. In the mammalian system the channel is formed by a central pore between five monomers, and the GABA-binding site is found at the interface of monomer subunits; perhaps a similar multimeric structure will be found for ALMTs as suggested by recent results from *At*ALMT9 [40].

## Conclusions

ALMTs have been shown to be involved in many vital roles in plants (summarized in Table 1), and there are many more likely to be found. For example, ten genes in *Arabidopsis* still have unassigned functions as seen in Figure 1. Some of these may have similar or redundant roles, for example *At*ALMT4 and *At*ALMT5 are closely related to *At*ALMT6 and so may also have a role in the guard cell vacuolar membrane. Indeed, some of these channels may account for residual activity seen in knockout mutants that still retain some function. Alternatively, ALMTs may form hetero-multimers to provide diversified functions, as seen in GABA(A) receptors in mammals. In addition, several members have expression patterns that show they must have a wider range of roles than is currently known–for example, *At*ALMT6 has a role in guard cells, but is also expressed in floral tissues with an as-yet undefined function. It is possible that ALMTs are involved in shuttling malate in C4 and CAM metabolism as the channels involved have not yet been identified. However, as *Arabidopsis* is a C3 plant these experiments will have to be done in another species.

Although there is a growing body of knowledge about the physiological functions of ALMTs, much less is known about their structure and mechanism. Relatively small differences in sequence can lead to large changes in localization, substrate specificity, gating and physiological function. In many cases, evidence is either scant or directly contradictory. No detailed 3D structure is available of any family member, but would help guide biochemical and functional studies and elucidate further details of mechanism and regulation, and thus detailed, high quality structural studies are vital for a full understanding ALMTs.

Finally, the ALMT family has been shown to have many members that are not aluminium activated, to have members permeable to anions other than malate, and to be channels rather than active transporters. Thus, the name aluminium-activated malate transporters does not fully reflect this family of proteins and is potentially confusing. It is perhaps advisable to take up the previously suggested QUAC nomenclature, which better reflects the characteristics of the family members.

## Abbreviations

Al: aluminium
ALMT: aluminium-activated malate transporter
CTD: C-terminal domain
GABA: γ-aminobutyric acid
QUAC: quick anion channel, R-type
SLAC: slow anion channels, S-type
